# Analysis and Prediction of Melt Pool Geometry in Rectangular Spot Laser Cladding Based on Ant Colony Optimization–Support Vector Regression

**DOI:** 10.3390/mi16020224

**Published:** 2025-02-16

**Authors:** Junhua Wang, Jiameng Wang, Xiaoqin Zha, Yan Lu, Kun Li, Junfei Xu, Tancheng Xie

**Affiliations:** 1 School of Mechanical and Electrical Engineering, Henan University of Science and Technology, Luoyang 471003, China; wangjh@haust.edu.cn (J.W.); wu31264234@163.com (J.W.); 2 National Key Laboratory of Marine Corrosion and Protection, Luoyang Ship Material Research Institute, Luoyang 471023, China; 3 School of Materials Science and Engineering, Henan University of Science and Technology, Luoyang 471023, China; luyan@haust.edu.cn; 4 State Key Laboratory of Mechanical Transmission for Advanced Equipment, Chongqing University, Chongqing 400044, China; kun.li@cqu.edu.cn; 5 College of Mechanical and Vehicle Engineering, Chongqing University, Chongqing 400044, China; mecha_xjf@163.com; 6 Henan Intelligent Manufacturing Equipment Engineering Technology Research Center, Luoyang 471003, China; 7 Henan Engineering Laboratory of Intelligent Numerical Control Equipment, Luoyang 471003, China

**Keywords:** wide beam laser cladding, melt pool width, melt pool area, ACO-SVR

## Abstract

The rectangular spot laser cladding system, due to its large spot size and high efficiency, has been widely applied in laser cladding equipment, significantly improving cladding’s efficiency. However, while enhancing cladding efficiency, the rectangular spot laser cladding system may also affect the stability of the melt pool, thereby impacting the cladding’s quality. To accurately predict the melt pool morphology and size during wide beam laser cladding, this study developed a melt pool monitoring system. Through real-time monitoring of the melt pool morphology, image processing techniques were employed to extract features such as the melt pool width and area. The study used laser power, scanning speed, and the powder feed rate as input variables, and established a prediction model for the melt pool width and area based on Support Vector Regression (SVR). Additionally, an Ant Colony Optimization (ACO) algorithm was applied to optimize the SVR model, resulting in an ACO-SVR-based prediction model for the melt pool. The results show that the relative error in predicting the melt pool width using the ACO-SVR model is less than 2.2%, and the relative error in predicting the melt pool area is less than 9.13%, achieving accurate predictions of the melt pool width and area during rectangular spot laser cladding.

## 1. Introduction

Laser cladding technology is an advanced, green, and efficient surface treatment technique. This technology utilizes high energy density lasers to rapidly melt metal powders on the surface of a substrate, forming a metallurgical bond with the substrate to create a surface reinforcement coating [1,2]. In recent years, with the rapid development of laser technology, rectangular spot, high-power wide-beam lasers have been widely applied in laser cladding equipment [3,4]. The rectangular spot wide-beam laser beam not only effectively improves the efficiency of laser cladding, but also significantly increases the intensity of physical and chemical reactions during the cladding process [5,6,7]. This, in turn, impacts the stability of the melt pool during the laser cladding process, which can affect the quality of the cladding. Additionally, the laser cladding process is complex and influenced by many factors [8,9]. A complicated nonlinear relationship exists between process parameters and melt pool morphology features. Therefore, constructing an accurate and reliable prediction model for melt pool morphology and size based on limited experimental data is fundamental for achieving high-quality laser cladding, making it of significant research importance.

In recent years, domestic and foreign scholars have carried out a lot of research on the monitoring and prediction of melt pool morphology in the laser cladding process, and some useful conclusions have been obtained. Bo Cheng et al. [10] studied the melt pool by using an infrared thermal imager to measure the cladding temperature. It was concluded that there was no significant difference in the melt pool length at different scanning speeds, while the scanning speed had a significant effect on the melt pool width. Shuang Liu et al. [11] used an infrared thermal imager to study the temperature distribution, melt pool size, and cooling rate under different process parameters. The results show that a change in the length of the melt pool is more significant than a change in the width, and that laser power and the carrier gas flow rate have the greatest influence on the size of the melt pool. As the laser power increases and the carrier gas flow rate decreases, the wake of the melt pool elongates. Aniruddha Gaikwad et al. [12] proposed an online monitoring method for laser cladding melt pool monitoring, based on a high-speed camera and melt pool temperature imaging system. By using data that represent multiple process phenomena rather than a single feature, the data fusion method significantly improves the system’s defect detection performance. Trong-Nhan Le et al. [13] developed a low-cost, high-speed off-axis monitoring system for measuring the width and length of the melt pool in the Laser Powder Bed Fusion process. The system uses a high-speed CMOS camera for image acquisition and realizes splash removal and size calculation through internal algorithms. The maximum errors are 18% and 15%, respectively, which proves the feasibility of the system. Song et al. [14] built a melt pool monitoring system using a side-axis high-speed camera, and proposed a phase-consistent melt pool edge extraction method, which has a better robustness to noise and interference, and can extract the geometric shape of the melt pool more accurately. Qi et al. [15] used the minimum circumscribed rectangle method to extract the width of the melt pool and used it as the feedback for the melt pool control. The experimental results show that the method can significantly improve the dimensional accuracy of the curved thin-walled workpiece. Luke Scime et al. [16] measured the cross-section of the melt pool under different process parameters to obtain information on the melt pool’s width, depth, and cross-sectional area, and conducted an in-depth analysis of the variation in these parameters. Vahid Fallah et al. [17] established a transient finite element model to simulate the temperature distribution and changes in the shape of the melt pool during laser powder deposition, demonstrating a good simulation capability for the deposition of Ti45Nb on Ti-6Al-4V. Niklas Sommer et al. [18] studied the influence of laser power and defocusing on the width of the cladding layer by observing the cross-section of the cladding.

However, at present, most of the research on the detection and prediction of the laser cladding melt pool’s morphology is concentrated on the circular spot, and there are few studies on predicting the width and area of the wide-beam laser cladding melt pool. The majority of these are monitored by infrared thermal imagers, and the monitoring cost is high. With the wide application of wide-beam laser cladding technology, it is urgent to construct a prediction model for melt pool morphology and size in wide-beam laser cladding to achieve high-precision predictive control of melt pool morphology in the wide-beam laser cladding process, so as to obtain high-quality laser cladding coatings.

In order to accurately predict the morphology and size of the melt pool in the process of wide-beam laser cladding, a monitoring device for the melt pool of wide-beam laser cladding was built in this study. The morphology of the melt pool was monitored in real time, and the width and area of the melt pool were extracted. Using laser power, scanning speed, powder feed rate, and other process parameters as input variables, a prediction model for melt pool width and area was established based on Support Vector Regression (SVR). Furthermore, the model was optimized using an Ant Colony Optimization (ACO) algorithm, significantly improving its prediction accuracy. This work provides theoretical guidance for the further application and promotion of wide-beam laser cladding technology.

## 2. Experimental Preparation

### 2.1. Experimental Equipment and Materials

**Figure 1:**
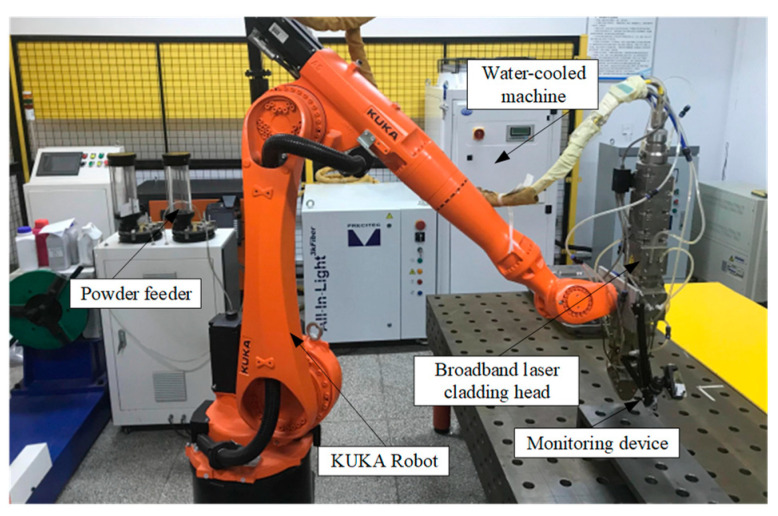
Wide-beam laser cladding system.

The wide-beam laser cladding system mainly includes a KUKA KR20 R1810-2 si*x*-axis robot system, an All-In-Light 3 kW fiber laser and water cooler, a wide-beam laser cladding head, and a JS-SFQ-02-2 carrier gas double-cylinder powder feeder. A physical diagram of the equipment is shown in Figure 1.

**Figure 2:**
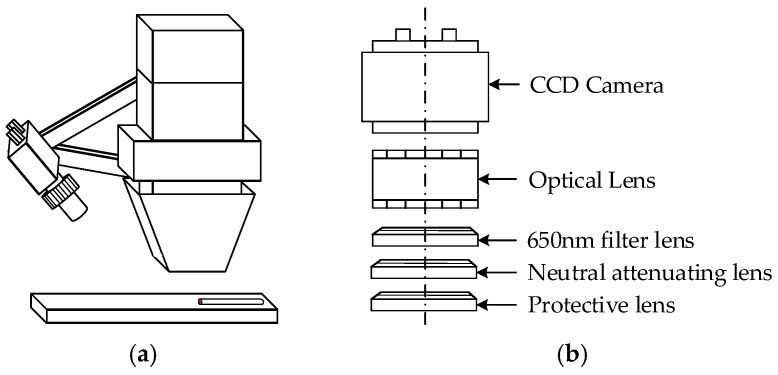
Melt pool monitoring device: (**a**) equipment installation schematic. (**b**) Filter components.

The melt pool monitoring device is mainly composed of a CCD camera, a 650 nm filter, and s 10% neutral attenuator which is installed at a 45° angle relative to the laser head. The installation schematic of the melt pool monitoring system and the positional relationship of the optical components are shown in Figure 2.

In the experiment, an H13 steel plate was chosen as the substrate material, with dimensions of 100 mm × 100 mm × 8 mm. Prior to the experiment, the substrate surface was ground, followed by washing with anhydrous ethanol and drying. The cladding material used was Inconel 718 alloy powder, which was dried before the cladding process.

### 2.2. Experimental Method and Design

**Figure 3:**
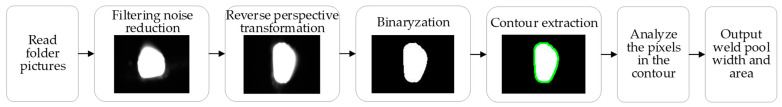
Image processing flow chart.

In this study, OpenCV was used to calibrate the industrial camera and process the images. The process of acquiring the width and area of the melt pool is shown in Figure 3. Real-time images of the melt pool were captured and saved by the CCD camera. The photos were then read sequentially for filtering and noise reduction, inverse perspective transformation, and binarization. The contour features of the melt pool were extracted, the number of pixel points within the melt pool contour was analyzed, and the width and area values of the melt pool were calculated by comparing them with the calibration values.

To accurately obtain the width and area of the melt pool in the images, precise calibration of the CCD camera is required. By obtaining the internal and external parameter matrices of the CCD camera, the conversion relationship between the pixel coordinates of the melt pool image and the actual spatial coordinates could be established. During measurement, the corresponding actual spatial coordinates were obtained by reverse-calculating the pixel coordinates in the image, thus providing an accurate size of the measured object in real space.

The orthogonal experimental method is an efficient experimental design technique that allows information about the influence of various factors to be obtained with fewer experimental runs. Since laser power, scanning speed, and powder feed rate are key process parameters that affect the melt pool morphology in wide-beam laser cladding [19], this study used laser power, scanning speed, and the powder feed rate as factors to design a 3-factor, 5-level L_25_(5^3^) orthogonal experiment to quantify their relationship with the melt pool width and area. Based on previous research conducted by our team, the range of parameters used in the experimental design was selected to cover the working range used in the actual production process as comprehensively as possible. The specific parameter settings are shown in Table 1.

## 3. Experimental Results

This study is based on a three-factor, five-level orthogonal experimental design, consisting of 25 experiments carried out under different process parameter combinations, with a single cladding pass length of 70 mm. To eliminate the interference of melt pool instability during the experiment, for each group of experiments, 1 image was sampled every 5 images from a set of 350 real-time melt pool images, resulting in a total of 70 image samples for processing. After obtaining the width and area values of the melt pool for each sample, the average was calculated and considered as the melt pool width and area under the given process parameters. Additionally, to quantify the stability of the melt pool under different process parameter combinations, the standard deviation of the melt pool width and area was calculated for each group of experiments. The calculation formula is as follows:

(1) $$L_{\sigma }=\frac{1}{n-1}{\sum_{i=1}^{n}\left(x_{1i}-{\bar{x}}_{1}\right)}^{2}$$

(2) $$S_{\sigma }=\frac{1}{n-1}{\sum_{i=1}^{n}\left(x_{2i}-{\bar{x}}_{2}\right)}^{2}$$

In the formula, $L_{\sigma }$ and $S_{\sigma }$ represent the standard deviations of the melt pool width and area, respectively; $x_{1i}$ and $x_{2i}$ represent the i-th observation values of the melt pool width and area, respectively; ${\bar{x}}_{1}$ is the average value of the melt pool width, ${\bar{x}}_{2}$ is the average value of the melt pool area, and n is the number of observation values in the sample.

The results of the melt pool width, area, and their standard deviations measured under different process parameter conditions are shown in Table 2.

Laser power is the primary determinant of the energy input into the melt pool. A higher laser power increases the matrix and powder melting, expanding the melt pool’s width and area. The scanning speed regulates energy distribution, with lower speeds prolonging the laser interaction, thereby enlarging the melt pool. The powder feeding speed controls the material supply during cladding, ensuring uniform layer formation. Deviations from the optimal feeding speed compromise cladding quality.

## 4. Prediction Model

### 4.1. SVR Model

To achieve accurate prediction of the melt pool morphology and size in wide-beam laser cladding under different process parameters, this study uses key process parameters such as laser power, scanning speed, and powder feed rate as input variables, with the melt pool width and area as output variables, to establish an SVR model. SVR can handle complex nonlinear relationships and is suitable for small datasets with significant noise [20,21]. The mathematical model of SVR is represented by Equation (3):

(3) $$f\left(x\right)=\omega^{T}\varphi (x)+b$$

where φ(x) is the feature space mapped by the kernel function, $\omega^{T}$ is the weight vector, *b* is the bias term, and $x_{i}\in R$ represents the input variables.

The objective of SVR is to minimize the complexity of and errors within the model, with the final optimization problem represented by Equation (4):

(4) $$R_{s}={\left[\frac{1}{2}{\left\|\omega \right\|}^{2}+C\sum_{i=1}^{n}\left(\xi_{i}+\xi_{i}^{*}\right)\right]}_{\min}$$

where $\xi_{i}$ and $\xi_{i}^{*}$ are slack variables, which represent the deviation of each sample point when it exceeds the insensitive loss function ε. *C* is the regularization parameter, which controls the trade-off between the error term and the model complexity. The larger *C* value will lead to more accurate fitting of the training data, but may lead to overfitting.

In order to ensure that the model does not over fit, the SVR is added with constraints:

(5) $$s.t=\left\{\begin{array}{l} y_{i}-f\left(x_{i}\right)\leq \xi_{i}+\varepsilon \\ f\left(x_{i}\right)-y_{i}\leq \xi_{i}^{*}+\varepsilon \\ \xi_{i},\xi_{i}^{*}\geq 0,i=1,2,\ldots ,n \end{array}\right.$$

These constraints produce non-zero slack variables $\xi_{i}$ and $\xi_{i}^{*}$ only when the error exceeds ε.

In this study, the rbf kernel function is selected, which is in the form of Equation (6). The rbf kernel function is a kernel function commonly used in SVR. It calculates the similarity between input data points using a Gaussian function, and maps the data to a high-dimensional feature space, so that the nonlinear problem can be solved by linear regression.

(6) $$K\left(x,x'\right)=\exp\left(-\frac{{\left\|x-x'\right\|}^{2}}{2\sigma^{2}}\right)$$

In order to simplify the formula, the parameter *γ* is used instead of $\sigma^{2}$, and the following formula (7) is obtained. This determines the ‘width’ of the kernel function and affects the data distribution after mapping to the feature space:

(7) $$K\left(x,x'\right)=\exp\left(-\gamma {\left\|x-x'\right\|}^{2}\right)$$

The 25 sets of test data were divided according to an 80% training set and a 20% test set. The radial basis function (rbf) is used as the kernel function, and the *γ* parameter is the ‘scale’, indicating that the default value is used. This setting helps to automatically adjust the value of *γ* to adapt to the characteristics of the data. The penalty coefficient *C* is 100, and the tolerance epsilon is 0.08. The normalization formula is shown in Equation (8):

(8) $$X^{'}=\frac{X-X_{\min}}{X_{\max}-X_{\min}}$$

The experimental data are first normalized, converting all data to the range [0, 1] to improve the stability and prediction accuracy of the model. *X* represents the original data, while *X*_max_ and *X*_min_ are the maximum and minimum values in the original data, respectively. *X’* represents the normalized data. After the normalization of all data, the model is trained using the dataset, and the SVR model is tested with the test set. The prediction accuracy of the model is evaluated by comparing the predicted values with the actual values of the experimental data. The true values of the width and area of the melt pool are shown in Table 3, where L represents the melt pool width, and S represents the melt pool area, L_y1_ and S_y1_ represent the width and area of the melt pool, respectively. The predicted value, Δ*_11_*, and Δ*_12_* represent the relative error between the predicted value and the experimental value of the width and area of the melt pool, respectively.

### 4.2. ACO-SVR Model

SVR has higher requirements for parameter adjustment, and appropriate adjustment and optimization are needed to obtain the best results. In this study, ACO is used to optimize the penalty coefficient *C* and the kernel function parameter *γ* of SVR.

ACO is an optimization algorithm that simulates the foraging behavior of ants and belongs to a group of bionic algorithms. It draws on the process of ants finding food through pheromone transmission [22,23]. In nature, ants secrete a chemical called pheromone on their path when looking for food, and the pheromone will gradually evaporate over time. Other ants will decide which path to choose according to the pheromone concentration on the path. The higher the pheromone concentration on the path, the greater the probability of selecting the path. Finally, the ant colony tends to choose the shortest path with the higher pheromone concentration.

After establishing feature data and label data, the data are divided according to an 80% training set and a 20% test set. In order to find the shortest path, it is necessary to initialize each parameter of the program: ant colony size m = 50; pheromone importance factor *α*; heuristic function importance factor *β*; pheromone volatilization factor; maximum iteration number ddcs_max = 50; initial iteration value ddcs = 1. Each ant is randomly placed at different starting points. At each step, the ant selects the next node *j* from the current node *i*, and its probability $P\left(i,j\right)$ is calculated using the following Formula (9):

(9) $$P\left(i,j\right)=\frac{{\left[\tau \left(i,j\right)\right]}^{\alpha }\cdot {\left[\eta \left(i,j\right)\right]}^{\beta }}{\sum_{k\in N_{i}}{\left[\tau \left(i,k\right)\right]}^{\alpha }\cdot {\left[\eta \left(i,k\right)\right]}^{\beta }}$$

where $\tau \left(i,j\right)$ is the pheromone concentration from node *i* to node *j*; $\eta \left(i,j\right)=\frac{1}{d\left(i,j\right)}$ is the heuristic function, which is the reciprocal of the distance from node *i* to node *j*; *α* is an important factor of the pheromone, which controls the influence of the pheromone on the ants’ selected path. *β* is the importance factor of heuristic information, which controls the influence of the reciprocal of distance on the selected path. *Ni* is the set of unvisited nodes adjacent to the current node *i*.

After the ants complete a round of searching, the pheromone needs to be updated according to the path quality. The goal of pheromone updating is to make the pheromone concentration on the path with better path quality higher, so that subsequent ants are more inclined to choose these paths. The pheromone update follows the following Formula (10):

(10) $$\tau \left(i,j\right)=\left(1-\rho \right)\cdot \tau \left(i,j\right)+\Delta \tau \left(i,j\right)$$

**Figure 4:**
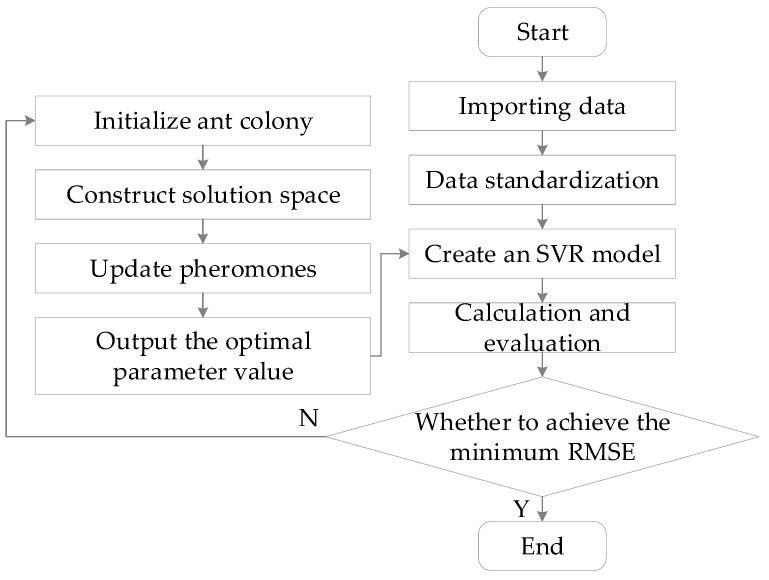
ACO-SVR model flow chart.

*ρ* is the pheromone volatilization factor, which controls the volatilization rate of the pheromone. $\tau \left(i,j\right)$ is the pheromone concentration updated by the contribution of all ants. A flow chart of the ACO-SVR model applied in this study is shown in Figure 4, and the SVR parameters optimized by ACO are shown in Table 4.

The prediction results and relative errors of the ACO-SVR model are shown in Table 5, where L_y2_ and S_y2_ represent the predicted values of the width and area of the melt pool, respectively, and Δ*_21_* and Δ*_22_* represent the relative errors between the predicted values of the width and area of the melt pool and the experimental values, respectively.

By analyzing the prediction results of the ACO-SVR model, it is found that the relative error of the melt pool width prediction is within 2.2%, and the relative error of the melt pool area prediction is within 9.13%.

### 4.3. Comparison and Analysis of Model Prediction Results

**Figure 5:**
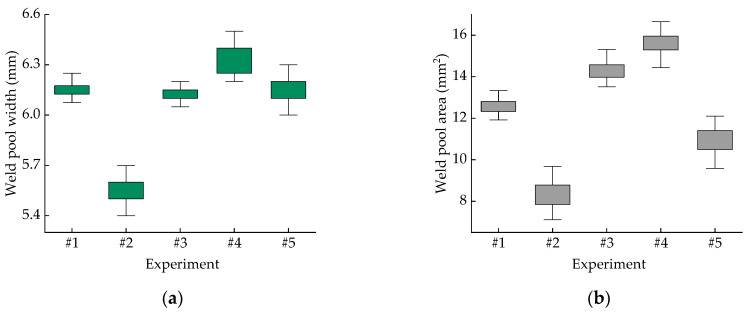
The experimental values of the melt pool width and area. (**a**) The width of the melt pool; (**b**) the area of the melt pool.

**Figure 6:**
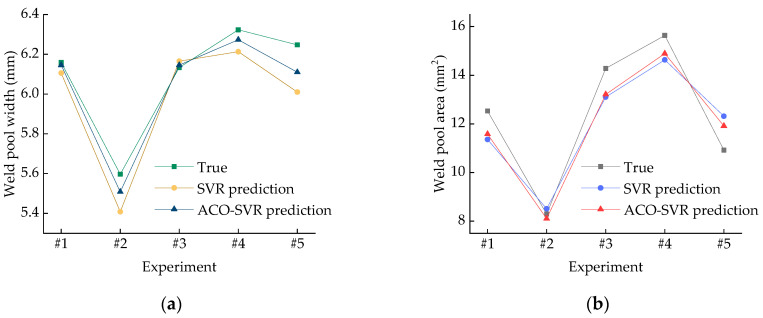
SVR model and ACO-SVR model. (**a**) Comparison of melt pool width prediction values; (**b**) comparison of melt pool area prediction values.

**Figure 7:**
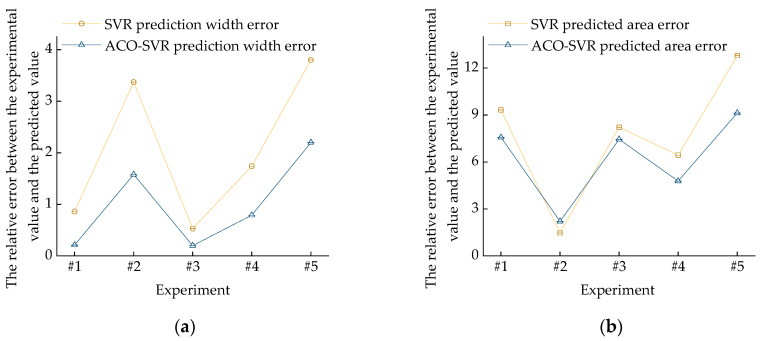
Relative errors: (**a**) comparison of relative errors between SVR and ACO-SVR melt pool width prediction values; (**b**) comparison of relative errors between SVR and ACO-SVR melt pool area prediction values.

The width and area of the melt pool, as measured by the monitoring device, are shown in Figure 5. A comparison of the prediction results between the SVR model and the ACO-SVR model is shown in Figure 6. The SVR predictions, optimized by the ACO algorithm, are more accurate. For the unoptimized SVR melt pool width prediction model, the relative error between the experimental and predicted values ranges from 0.53% to 3.80%, while for the SVR melt pool area prediction model, the relative error between the experimental and predicted values ranges from 1.46% to 12.79%. For the optimized ACO-SVR melt pool width prediction model, the relative error between the experimental and predicted values ranges from 0.20% to 2.20%, and for the melt pool area prediction model, the relative error ranges from 2.21% to 9.13%. A comparison of the relative errors between the SVR and ACO-SVR models for the melt pool width and area predictions is shown in Figure 7. Based on an analysis of the model characteristics, it is concluded that the traditional SVR model requires manual selection of hyperparameters, such as kernel function type and penalty coefficient, while ACO can more quickly identify the optimal parameter settings that allow the SVR model to achieve its best performance. These characteristics help to find a better combination of model parameters, thereby improving the accuracy of the SVR prediction model. The width and area of the melt pool directly influence the geometric accuracy and surface quality of the workpiece. Enhancing the prediction accuracy of the melt pool width and area helps to ensure consistency in the workpiece’s size, shape, and material properties. ACO-SVR combines ACO and SVR, both of which have a high computational complexity and require long computational times. In future research, heuristic rules can be explored to reduce unnecessary search spaces.

## 5. Conclusions

In this study, the characteristics of the width and area of the melt pool in the process of wide beam laser cladding were extracted by building a melt pool monitoring system. Based on ACO-SVR, the prediction models of the width and area of the melt pool were established, respectively, and the following conclusions were obtained:

(1) The changes in process parameters such as laser power, scanning speed, and powder feeding rate will affect the morphology and size of the melt pool in wide beam laser cladding. Different combinations of process parameters correspond to different morphologies and sizes of the melt pool.
(2) The prediction models for melt pool width and area in wide-beam laser cladding, established using the SVR, can effectively predict the melt pool width and area, with relative errors less than 3.80% and 12.79%, respectively. However, these errors are relatively large and require further optimization.
(3) The penalty coefficient *C* and kernel function parameter *γ* of the SVR model of melt pool width and area are optimized using the ant colony algorithm. The relative error of the ACO-SVR melt pool width prediction model was less than 2.20%, while the relative error of the melt pool area prediction model was less than 9.13%.

## Figures and Tables

**Table 1:** Orthogonal experimental design for factor levels and testing.

Level Figure	Factor		
	Laser Power P (W)	Scanning Speed V (mm/s)	Powder Feed Rate N (r/min)
#1	1500	4	4
#2	1750	8	6
#3	2000	12	8
#4	2250	16	10
#5	2500	20	12

**Table 2:** Results of orthogonal test.

NO.	P (W)	V (mm/s)	N (r/min)	Width (mm)	Area (mm^2^)	Width Standard Deviation	Area Standard Deviation
#1	1500	4	4	6.16	12.53	0.0497	0.3515
#2	1500	8	6	5.85	9.37	0.0663	0.2305
#3	1500	12	8	5.59	7.96	0.1073	0.3597
#4	1500	16	10	5.37	6.53	0.1373	0.3855
#5	1500	20	12	5.01	4.67	0.1866	0.3830
#6	1750	4	6	6.18	13.10	0.0493	0.2382
#7	1750	8	8	6.03	11.64	0.1005	0.2179
#8	1750	12	10	5.80	9.70	0.1067	0.3373
#9	1750	16	12	5.60	8.29	0.1397	0.5071
#10	1750	20	4	5.54	6.97	0.1388	0.5100
#11	2000	4	8	6.50	17.37	0.0902	0.3793
#12	2000	8	10	6.13	14.28	0.1086	0.2362
#13	2000	12	12	5.91	11.60	0.1139	0.2183
#14	2000	16	4	5.99	10.29	0.1598	0.2839
#15	2000	20	6	5.70	7.74	0.1672	0.4442
#16	2250	4	10	6.63	20.06	0.0824	0.3305
#17	2250	8	12	6.32	15.64	0.0765	0.3252
#18	2250	12	4	6.31	12.67	0.2199	0.2115
#19	2250	16	6	6.03	10.38	0.1946	0.3590
#20	2250	20	8	5.80	8.50	0.0984	0.5159
#21	2500	4	12	6.76	22.09	0.1061	0.6017
#22	2500	8	4	6.44	14.18	0.1325	0.2421
#23	2500	12	6	6.27	12.56	0.1986	0.3185
#24	2500	16	8	6.25	10.92	0.2811	0.3742
#25	2500	20	10	5.96	9.45	0.2761	0.4888

**Table 3:** Width and area of melt pool.

NO.	P (W)	V (mm/s)	N (r/min)	L (mm)	L_y1_ (mm)	Δ*_11_* (%)	S (mm^2^)	S_y1_ (mm^2^)	Δ*_12_* (%)
#1	1500	4	4	6.16	6.11	0.86	12.53	11.36	9.33
#2	1750	16	12	5.60	5.41	3.37	8.29	8.41	1.46
#3	2000	8	10	6.13	6.17	0.53	14.28	13.81	3.32
#4	2250	8	12	6.32	6.21	1.74	15.64	14.63	6.44
#5	2500	16	8	6.25	6.01	3.80	10.92	12.32	12.79

**Table 4:** SVR model parameters.

	*C*	*γ*
melt pool width	1.000229	0.008837
melt pool area	1.001707	0.025461

**Table 5:** The predicted value of the melt pool width and area.

NO.	L (mm)	L_y2_ (mm)	Δ*_21_* (%)	S (mm^2^)	S_y2_ (mm^2^)	Δ*_22_* (%)
#1	6.16	6.15	0.21	12.53	11.58	7.58
#2	5.60	5.51	1.58	8.29	8.11	2.21
#3	6.13	6.15	0.20	14.28	13.22	7.45
#4	6.32	6.27	0.79	15.64	14.89	4.78
#5	6.25	6.11	2.20	10.92	11.92	9.13

## Data Availability

Data are contained within the article.
